# Contrasting Effects of Elevated Temperature and Invertebrate Grazing Regulate Multispecies Interactions between Decomposer Fungi

**DOI:** 10.1371/journal.pone.0077610

**Published:** 2013-10-23

**Authors:** A. Donald A′Bear, William Murray, Rachel Webb, Lynne Boddy, T. Hefin Jones

**Affiliations:** Cardiff School of Biosciences, Cardiff University, Cardiff, United Kingdom; Portland State University, United States of America

## Abstract

Predicting the influence of biotic and abiotic factors on species interactions and ecosystem processes is among the primary aims of community ecologists. The composition of saprotrophic fungal communities is a consequence of competitive mycelial interactions, and a major determinant of woodland decomposition and nutrient cycling rates. Elevation of atmospheric temperature is predicted to drive changes in fungal community development. Top-down regulation of mycelial growth is an important determinant of, and moderator of temperature-driven changes to, two-species interaction outcomes. This study explores the interactive effects of a 4 °C temperature increase and soil invertebrate (collembola or woodlice) grazing on multispecies interactions between cord-forming basidiomycete fungi emerging from colonised beech (*Fagus sylvatica*) wood blocks. The fungal dominance hierarchy at ambient temperature (16 °C; *Phanerochaete velutina* > *Resinicium bicolor* > *Hypholoma fasciculare*) was altered by elevated temperature (20 °C; *R. bicolor* > *P. velutina* > *H. fasciculare*) in ungrazed systems. Warming promoted the competitive ability of the fungal species (*R. bicolor*) that was preferentially grazed by all invertebrate species. As a consequence, grazing prevented the effect of temperature on fungal community development and maintained a multispecies assemblage. Decomposition of fungal-colonised wood was stimulated by warming, with implications for increased CO_2_ efflux from woodland soil. Analogous to aboveground plant communities, increasing complexity of biotic and abiotic interactions appears to be important in buffering climate change effects on soil decomposers.

## Introduction

The influence of environmental change on species interactions is a major determinant of community structure and function [1]. Predicting how biotic and abiotic context affects the responses of species interactions and ecosystem processes to climate change remains one of the greatest challenges in community ecology [2]. Though the key determinants of plant community dynamics have been extensively explored (e.g. [3]–[5]), little is known about belowground community responses to climate change. While the importance of soil microbial communities in carbon and nutrient cycling is widely acknowledged [6], [7], the mechanisms regulating their composition and functioning remain poorly resolved. Fungi, in particular, exert a strong influence on soil organic matter dynamics and the temperature-sensitivity of decomposition [8]. The production of lignocellulolytic enzymes by saprotrophic basidiomycete fungi makes them primary regulators of decomposition in temperate woodland ecosystems [9], [10]. Filamentous networks of mycelium formed by a major ecological grouping of these fungi, the saprotrophic cord-formers, ramify at the soil–litter interface where they forage for woody resources [11], [12]. Competitive mycelial interactions determine fungal dominance and community composition [13]. These fungi largely compete, for space first and foremost, by antagonistic mechanisms commonly referred to as combat [13]. Factors affecting competitive fungal interaction outcomes will influence nutrient cycling and CO_2_ efflux from soil, as a consequence of species-specific extracellular enzyme production and resource decomposition rates [14], [15].

Temperature is an important abiotic determinant of decomposer fungal growth and activity [16]. Differential warming-induced stimulation of competing saprotrophic basidiomycete mycelial growth and combative ability has been shown to alter interaction outcomes in soil [17], [18]. During mycelial interactions, elevated temperature only increases the growth of the dominant competitor; increased growth by the inferior competitors is prevented by antagonism (overgrowth or direct killing of mycelia) during combative interactions [17]. Warming may also indirectly influence the biotic pressures exerted on fungal interactions by driving changes in the wider soil community [14], [19]. As a component of the first trophic level in the decomposer food web, soil fungi support a vast abundance of invertebrates [20]. Although soil invertebrate grazers are known to limit the growth of individual mycelial systems [16], [21] and, by feeding selectively on different species, alter the relative abundance of co-existing litter fungi [22], [23], only recently has selective grazing strong enough to reverse fungal dominance been revealed [17], [24]. Invertebrate macrofauna, particularly woodlice, exert the most consistent influences on individual and interacting mycelia [24], [25]. The potential for mycophagous collembola (mesofauna) to influence fungal growth and competitive dominance is determined by grazing intensity [17], [26]–[28].

The influences of elevated temperature and grazing by a range of soil invertebrate taxa on the outcomes of two-species interactions between three common species of temperate woodland decomposer fungi (*Hypholoma fasciculare*, *Phanerochaete velutina* and *Resinicium bicolor*) have been determined (Table 1). The capacity of grazing to alter fungal dominance and responses to abiotic change has implications for top-down regulation of saprotrophic basidiomycete community development. It is, however, unclear whether the fungal dominance hierarchy, and roles of biotic and abiotic factors, apparent from two-species interactions would be sustained or altered (potentially becoming weaker or more idiosyncratic) in more complex assemblages. This study explored the interactive effects of soil invertebrate (collembola or woodlice) grazing and warming on the outcomes of three-species interspecific mycelial interactions and fungal-mediated wood decomposition.

**Table 1:** Effects of temperature and grazing on the outcomes of two-species interactions between *Hypholoma fasciculare*, *Phanerochaete velutina* and *Resinicium bicolor*.

Interaction	Temperature (°C)	Grazer	Dominant fungus	Reference
*P. velutina* vs. *R. bicolor*	15	Ungrazed	*P. velutina*	[17]
	16		Draw	[18]
	18, 20		*R. bicolor*	[18], [24]
	15	*Folsomia candida* (Collembola)	Mixed	[17]
	18		*P. velutina*	[24]
	20		*R. bicolor*	
		*Panagrellus redivivus* (Nematoda)	*R. bicolor*	
		*Blaniulus guttulatus* (Diplopoda)	*R. bicolor*	
		*Oniscus asellus* (Isopoda)	*P. velutina*	
	16, 20	*O. asellus* (restricted to *P. velutina*)	Draw	[18]
	16	*O. asellus* (restricted to *R. bicolor*)	*P. velutina*	
	20		Draw	
*H. fasciculare* vs. *R. bicolor*	15	Ungrazed	Mixed	[17]
	16		*R. bicolor*	[18]
	18		*H. fasciculare*	[17]
	20		*R. bicolor*	[18], [24]
	15, 18	*F. candida*	*H. fasciculare*	[17]
	20		Draw/*R. bicolor*	[24]
		*P. redivivus*	Draw/*H. fasciculare*	
		*B. guttulatus*	*R. bicolor*	
		*O. asellus*	*H. fasciculare*	
		*O. asellus* (restricted to either fungus)	Draw	[18]
*H. fasciculare* vs. *P. velutina*	15, 16, 18, 20	Ungrazed, *F. candida*, *P. redivivus*, *B. guttulatus*, *O. asellus*	*P. velutina*	[17], [18], [24]
	16	*O. asellus* (restricted to *P. velutina*)	Draw/*P. velutina*	[18]
	20		*P. velutina*	
	16	*O. asellus* (restricted to *H. fasciculare*)	Draw/*P. velutina*	
	20		Draw	

“Draw”: neither competing fungus became dominant.

“Mixed”: all possible outcomes (win by both fungi and draw) were recorded.

A microcosm approach was employed to overcome limitations to mechanistic understanding of species interactions imposed by the complex and opaque nature of the soil decomposer system [29], [30]. The experimental system is a good model of cord-forming basidiomycete ramification at the soil–litter interface, where their mycelia come into direct contact with invertebrate fauna inhabiting soil humus and litter layers. All fungus and invertebrate species used in the study are common in temperate woodland soil. Communities containing these fungal species develop in temperate woodland, where they have been isolated from the decaying wood and litter of several tree species; over time they compete and replace one another within these resources [31], [32]. The study aimed to determine: (I) the influence of elevated temperature on fungal dominance and wood decay in three-species interactions; and (II) the extent to which grazing invertebrates moderate the influence of temperature on interaction outcomes and decomposition. Three specific hypotheses were tested: (I) elevated temperature will alter the fungal dominance hierarchy in ungrazed controls; (II) invertebrate grazing will alter competitive interaction outcomes and moderate the influence of elevated temperature; and (III) mycelial growth (measured as extension rate) and wood decay by the dominant competitor will be stimulated by warming.

## Materials and Methods

### Experimental design

The influence of soil invertebrate grazing (*Folsomia candida* and *Protophorura armata*, Collembola; *Oniscus asellus*, Isopoda) on the outcomes of three-way competitive interactions between cord-forming basidiomycete fungi (*Hypholoma fasciculare*, *Phanerochaete velutina* and *Resinicium bicolor*), at ambient and elevated (ambient +4 °C) temperature, was investigated in compressed soil microcosms. The temperature elevation is within the rise of 1.1 – 6.4 °C predicted for temperate regions by 2100 [33]. Organisms living within soil are not expected to experience the same extent of climate warming as will occur in the atmosphere. The soil–litter interface (where the interactions considered in the present study occur), however, will provide little buffering against atmospheric temperature change [34]. Ambient temperature (16 °C) represented late summer – autumn temperatures beneath the litter layer in UK temperate woodland [35]. A fully-factorial experimental design including ungrazed controls was employed, with 5 – 6 replicates for all treatments.

### Microcosm preparation

Topsoil from mixed deciduous woodland (Tintern, UK, NGR 352800 201800; 51.711, −2.684; permission issued by the UK Forestry Commission) was sieved through a 10 mm mesh on site and air-dried in the laboratory. Dry soil was then sieved through a 2 mm mesh, frozen for 24 h (to prevent population explosions of endogenous collembola; [16]) and mixed with de-ionised water (DH_2_O; 340 ml kg^−1^). Microcosms consisted of lidded clear plastic dishes (24 × 24 cm, 2 cm deep; Nunc-Gibco, Paisley, UK) containing 200 g of soil, which was evenly compressed to ensure fungi grew on the surface of the soil. This enabled visual quantification of mycelial growth and interpretation of interaction outcomes [17], [18], [24]. Freshly-felled beech (*Fagus sylvatica*) wood was cut into blocks (2 × 2 × 1 cm) and autoclaved three times at 24 h intervals. Sterile wood blocks were placed on 2% malt agar pre-colonised by the experimental fungi and incubated for 3 months in darkness at 16±1°C.

Collembola populations (Cardiff University Culture Collection) were maintained on a medium consisting of 95% plaster of Paris (Minerva Dental, Cardiff, UK) and 5% activated charcoal (Sigma, Poole, UK), in darkness at 20 ± 1 °C. Collembola were fed dried baker's yeast (*Saccharomyces*; Spice of Life, Cardiff, UK), and plaster was re-moistened with DH_2_O, weekly. Prior to experimental use, collembola were size-selected (200–400 µm body width) using a series of stacked sieves (Nickel-Electro Ltd, Weston-super-Mare, UK). Adult *O. asellus* (≥ 5 mm body length) were collected from Cooper's Field, Cardiff, UK (NGR 317819 176785; 51.487, −3.185; permission issued by Cardiff Council). Invertebrates were deprived of food for 24 h prior to addition to microcosms.

Colonised wood blocks were placed 8 cm apart in an equilateral triangular arrangement. Inoculation was staggered (order: *R. bicolor*, *H. fasciculare*, then *P. velutina*) according to species-specific mycelial emergence and extension rates, to ensure mycelia were of equal size when they met [24], [25]. This ensured that any treatment effects were a result of temperature or grazing regime and not the relative sizes (which could influence competitive ability) of interacting mycelia. Microcosms were weighed and sprayed weekly with DH_2_O to replace moisture loss. Once opposing mycelia had met (8 cm diameter) in 50% of microcosms, at each temperature (16 and 20 °C), 60 *F. candida*, 73 *P. armata* (to provide equal collembola biomass) or 5 *O. asellus* were added in grazing treatments. Invertebrate numbers represented low field density estimates [36], [37]. Microcosms that were not in the required standardised condition prior to grazer addition and experimental monitoring (e.g. one of the mycelia failed to emerge from the wood block) were discarded, leaving 5 replicates for all treatment combinations except grazed systems at ambient temperature, which each had 6.

### Data collection and analysis

A fungus was considered to gain territory on soil if it directly killed extra-resource mycelium, or overgrew and halted the growth, of an opponent. To gain territory in wood, a fungus had to reach an opponent's wood block and replace the original inhabitant. Wood blocks that had been reached by an opposing species were cut in half and wood chips (approx. 5 × 2 × 2 mm) removed to determine occupancy. Wood chips were dipped in 5% bleach (to prevent contamination), placed on 2% malt agar and incubated in darkness at 16 ± 1 °C. The mycelia that emerged were identified visually and only one species was ever isolated from each wood block. Differences in the frequencies of four possible interaction outcomes (*H. fasciculare*, *P. velutina* or *R. bicolor* ‘gain territory’, or ‘draw’ if no fungus gained territory), on soil and in wood blocks, between (I) grazing treatments at each temperature, and (II) temperatures for each grazing treatment, were assessed using binomial (two outcomes) or multinomial (three or four outcomes) logistic regression (Minitab 16).

Digital images were captured (Nikon Coolpix 7500 camera; 39.5 cm height under normal laboratory lighting) 0, 2, 6, 12, 20, 30 and 42 d after grazer addition to microcosms. Mycelial extent of each fungus was measured as the mean length of four lines from the wood block centre to the mycelial margin, through a 60° angle towards the opposing fungi (ImageJ; National Institute of Health, USA). Measurement ceased for a species when the first individual in any treatment reached an opposing wood block. Extension rates (cm d^−1^) of each fungus were analysed using two-way analysis of variance (ANOVA) and planned contrasts (R version 2.15.1; [38]), with grazing treatment and temperature as factors.

The state of decay (density, fresh volume/dry weight; g cm^−3^) of each wood block was determined at the end of the experiment (42 d). Decay rates (g cm^−3^ d^−1^) were estimated after determining pre-treatment density of additional wood blocks (n = 5) from which the relevant fungal species had emerged, at the relevant temperature. Decay rates were analysed using a three-way ANOVA and planned contrasts (R version 2.15.1; [38]), with fungal species, grazing treatment and temperature as factors.

## Results

### Interaction outcomes

**Figure 1:**
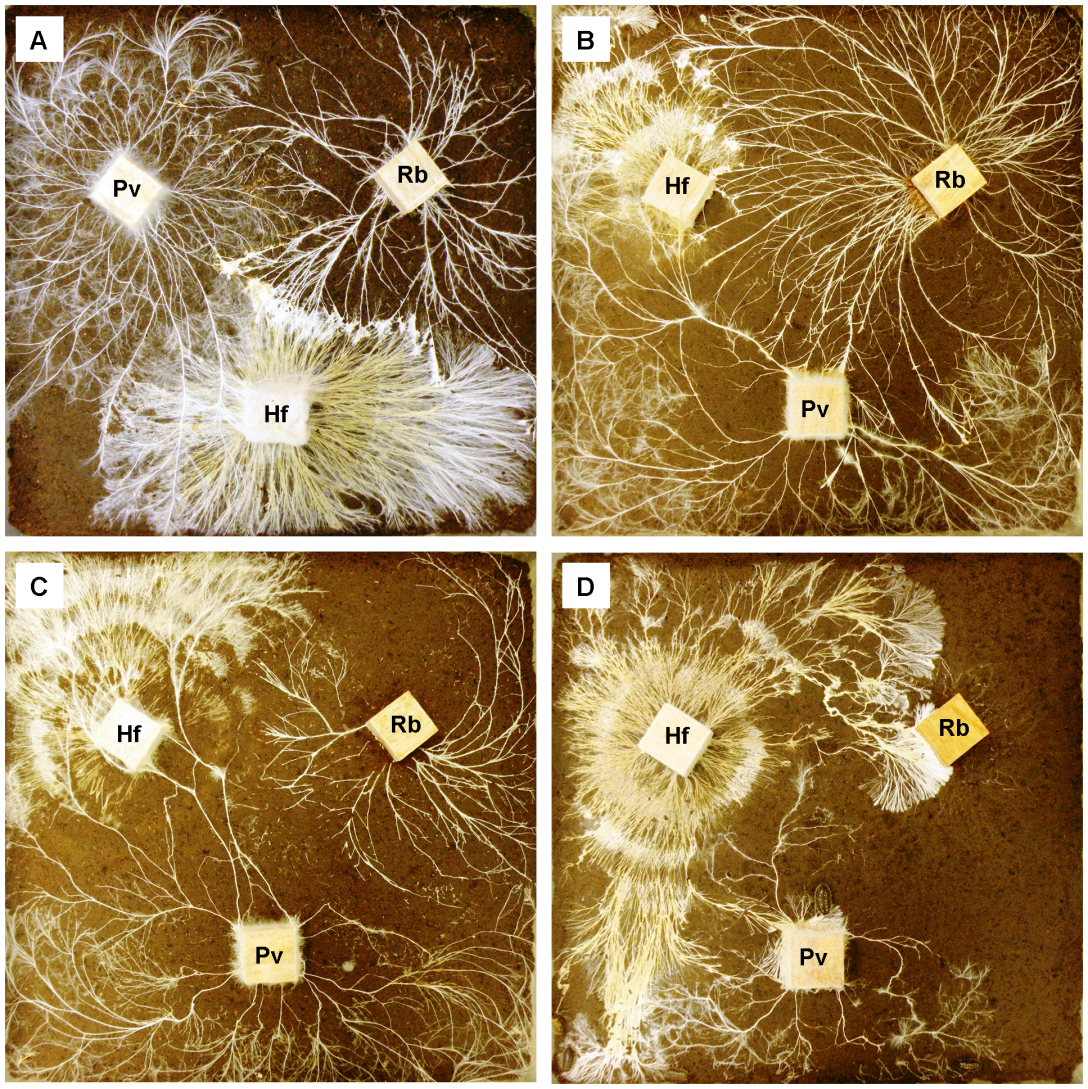
Digital images of mycelial interactions between *Hypholoma fasciculare* (Hf), *Phanerochaete velutina* (Pv) and *Resinicium bicolor* (Rb) growing from *Fagus sylvatica* wood blocks in soil microcosms. Treatments are: (A) ungrazed control at ambient temperature (16 °C); (B) ungrazed control, (C) *Folsomia candida*- and (D) *Oniscus asellus*-grazed at elevated temperature (20 °C). Wood block edges are 2 cm.

**Figure 2:**
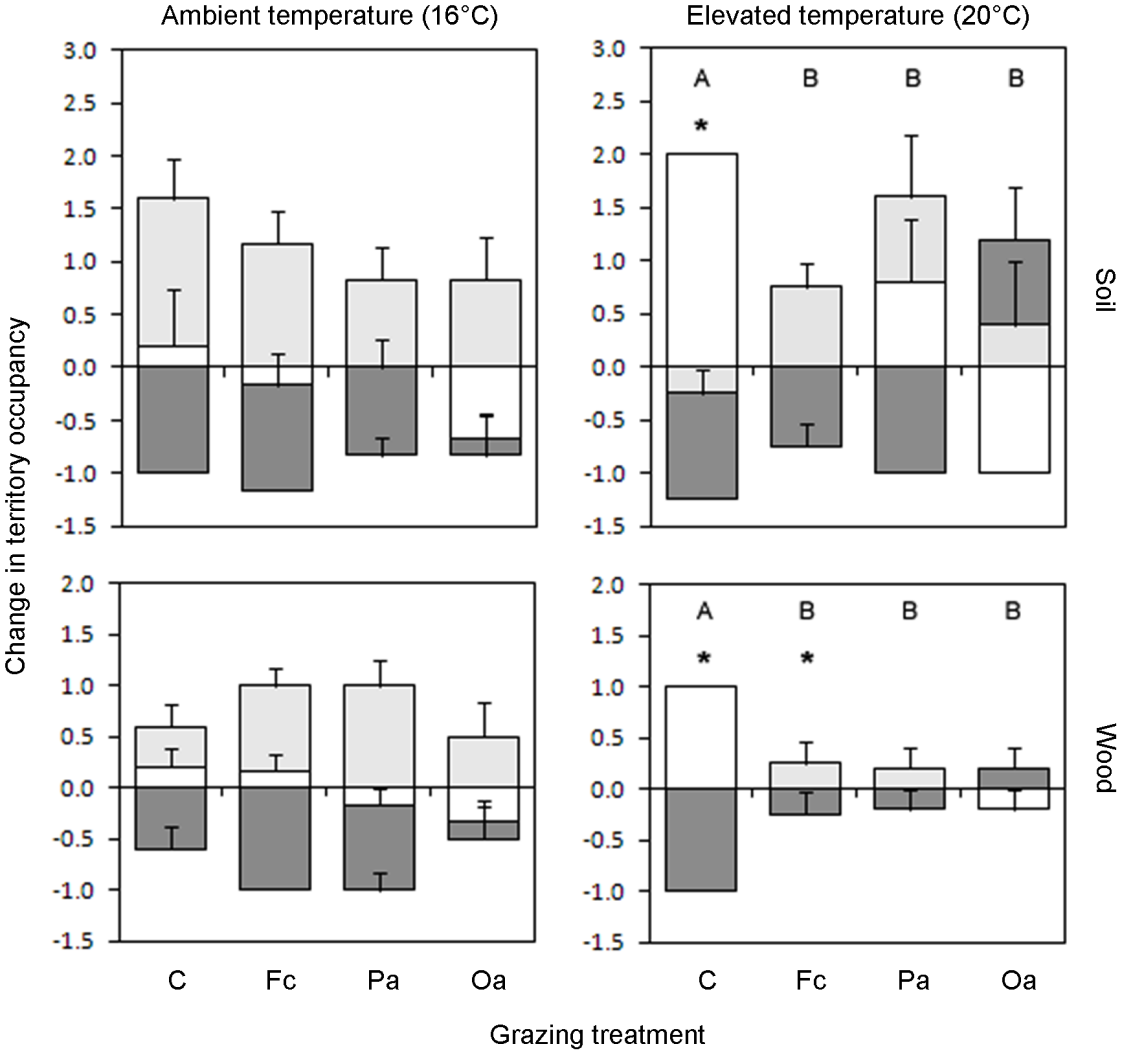
Change in territory occupancy (mean number of territories lost or gained ± SEM) on soil and in *Fagus sylvatica* wood blocks during three-way mycelial interactions between *Hypholoma fasciculare* (dark shading), *Phanerochaete velutina* (light shading) and *Resinicium bicolor* (unshaded), at ambient (16 °C) and elevated (20 °C) temperature. Each individual fungus could only lose one territory (that which it originally occupied), but had the potential to gain up to two if it replaced both competitors. Size of shaded areas, rather than total heights of the bars, indicate the magnitude of change. Significant (P<0.05) differences in interaction outcomes between grazing treatments are indicated by different letters. * indicates where the interaction outcome at elevated temperature differs significantly (P<0.05) from ambient temperature for the same grazing treatment.

In ungrazed systems, clear hierarchies of competitive dominance were evident, and differed between ambient (*P. velutina* > *R. bicolor* > *H. fasciculare*) and elevated (*R. bicolor* > *P. velutina* > *H. fasciculare*) temperature (Fig 1a, b; Fig 2; soil: G_1_ = 7.4, P = 0.007; wood: G_2_ = 7.4, P = 0.025). Where *P. velutina* was dominant it overgrew competitors (Fig 1a) and often replaced them in wood (Fig 2). Where *R. bicolor* was dominant it directly killed competing mycelia as it extended (Fig 1b). At elevated temperature, *P. velutina* usually gained territory from *H. fasciculare*, but lost its original territory to *R. bicolor* in ungrazed controls (Fig 1b).

All invertebrates preferentially consumed *R. bicolor* and avoided *H. fasciculare*, except in areas of combative interaction. As a consequence, grazing altered interaction outcomes at elevated temperature (Fig. 1 b, c, d; Fig. 2; soil: G_10_ = 22.3, P = 0.008; wood: G_10_ = 24.1, P = 0.004). *Oniscus asellus* rapidly removed the entire mycelial coverage of *R. bicolor*, after which they also grazed *P. velutina*; this enabled *H. fasciculare* to reach opposing wood blocks in some replicates (Fig. 1d; Fig. 2). Grazing significantly (P<0.05) reduced the change in territory occupancy in wood at elevated temperature (Fig. 2).

### Mycelial extension

**Figure 3:**
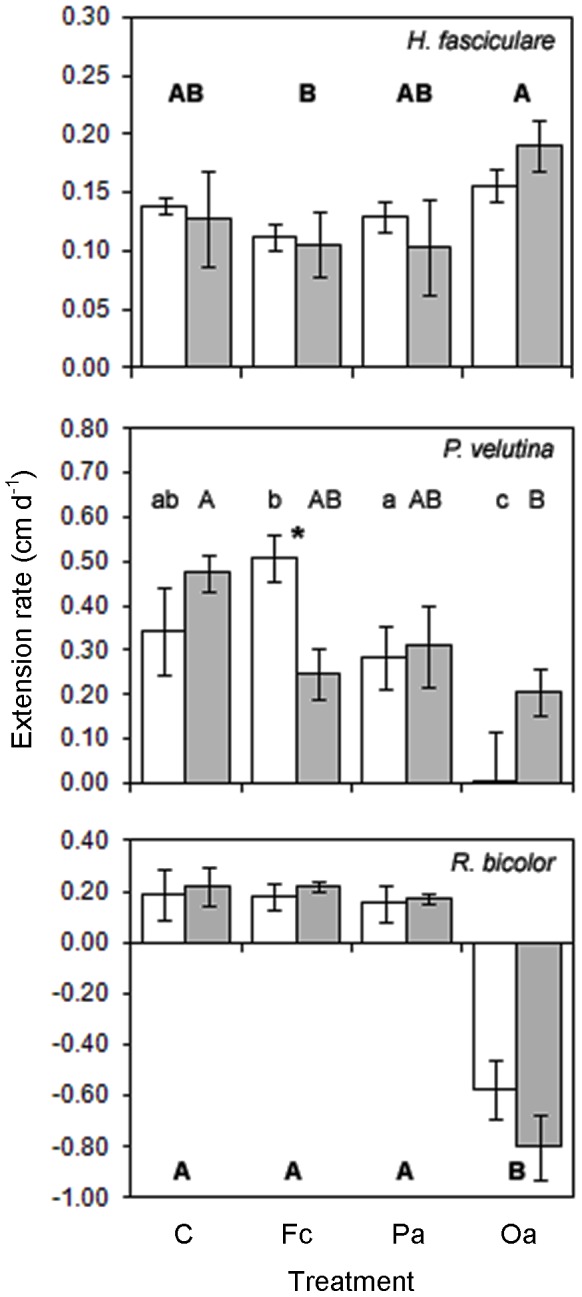
Extension rates (mean ± SEM) of *Hypholoma fasciculare*, *Phanerochaete velutina* and *Resinicium bicolor* during three-way mycelial interactions at ambient (16 °C; unshaded bars) and elevated (20 °C; shaded bars) temperature, in the ungrazed control (C), and *Folsomia candida* (Fc), *Protophorura armata* (Pa) and *Oniscus asellus* (Oa) grazing treatments. Different bold uppercase letters indicate significant (P<0.05) differences between grazing treatments across both temperatures. Different non-bold lowercase and uppercase letters indicate significant (P<0.05) differences between grazing treatments at ambient and elevated temperature, respectively. * indicates significant (P<0.05) differences between ambient and elevated temperature, for a given grazing treatment. Y axes differ between graphs.

Grazing treatment significantly affected mycelial extension rates of all fungi (Table 2; Fig. 3). *Oniscus asellus* grazing reduced *P. velutina* (P = 0.004) extension and reversed that of *R. bicolor* (P<0.001), enabling *H. fasciculare* to extend more rapidly and at a significantly (P = 0.030) greater rate than in the *F. candida* treatment (Fig. 3). Elevated temperature did not significantly (P ≥ 0.05) affect extension of any fungus overall, but reduced that of *P. velutina* only when grazed by *F. candida* (grazer*temperature: Table 2; Fig. 3).

**Table 2:** Overall effects (two-way ANOVA: F_degrees of freedom_ and P values) of temperature, grazing and their interaction of mycelial growth rates of *Hypholoma fasciculare*, *Phanerochaete velutina* and *Resinicium bicolor*.

	*H. fasciculare*		*P. velutina*		*R. bicolor*	
	F_3, 37_	P	F_3, 37_	P	F_3, 37_	P
Temperature						
Grazing	3.4	0.030	6.1	0.002	54.7	<0.001
Temperature × Grazing			3.2	0.034		

Non-significant (P ≥ 0.05) results are omitted for clarity.

### Wood decay rates

**Figure 4:**
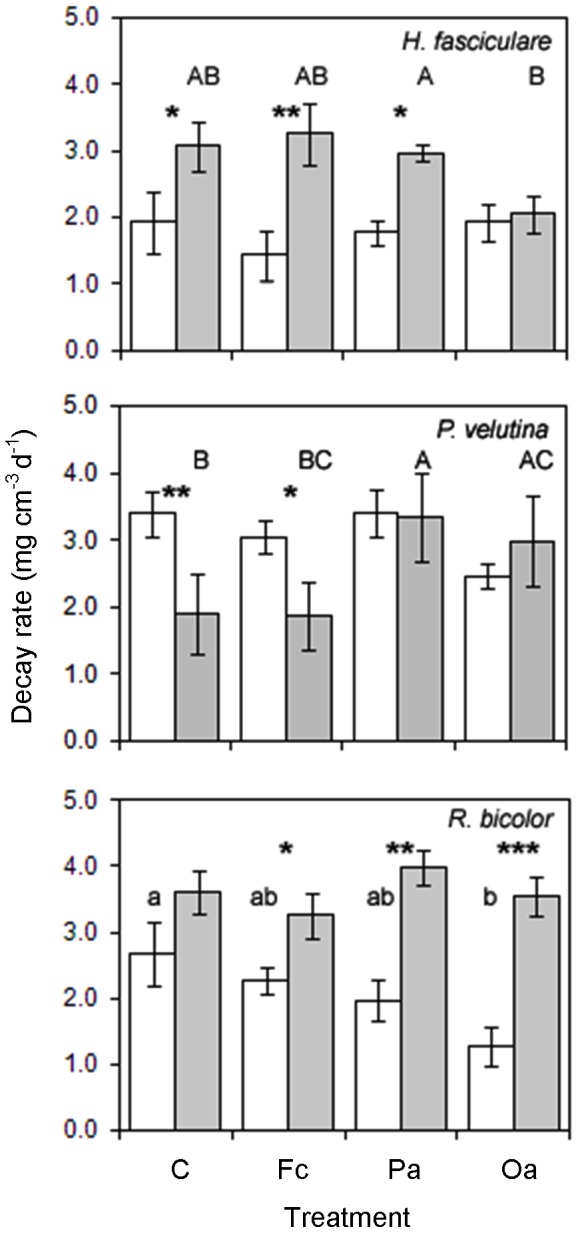
Decay rates (mean ± SEM) of *Fagus sylvatica* wood blocks colonised by *Hypholoma fasciculare*, *Phanerochaete velutina* and *Resinicium bicolor* during three-way mycelial interactions at ambient (16 °C; unshaded bars) and elevated (20 °C; shaded bars) temperature, in the ungrazed control (C), and *Folsomia candida* (Fc), *Protophorura armata* (Pa) and *Oniscus asellus* (Oa) grazing treatments. Different lowercase and uppercase letters indicate significant (P<0.05) differences between grazing treatments at ambient and elevated temperature, respectively. Significant differences between ambient and elevated temperature for a given grazing treatment are indicated (* P<0.05, ** P<0.01, *** P<0.001).

Decay rates differed between fungi (Table 3); both *P. velutina* (P = 0.004) and *R. bicolor* (P = 0.009) decayed wood more rapidly than *H. fasciculare*. Although warming did increase decay rates, overall (Table 3), this was driven by the effects on *H. fasciculare*- (P<0.001) and *R. bicolor*- (P<0.001) mediated decay (temperature*fungus: Table 3; Fig. 4). In contrast, warming reduced decay by *P. velutina* (P = 0.043; Fig. 4). Impacts of grazing on fungal-mediated decay differed between species and were altered by warming (grazer*temperature*fungus: Table 3; Fig. 4). Grazing influenced *H. fasciculare*- and *P. veluatina*-mediated decay only at elevated temperature, and *R. bicolor*-mediated decay only at ambient temperature (Fig. 4). *Oniscus asellus* grazing prevented warming from stimulating decay by *H. fasciculare*, whereas all grazers intensified the effect of warming on decay by *R. bicolor* (Fig. 4). Grazing by *P. armata* and *O. asellus* increased decay by *P. velutina* at elevated temperature and prevented warming-induced reduction in decay, which was evident in control and *F. candida*-grazed treatments (Fig. 4).

**Table 3:** Overall effects (three-way ANOVA: F, degrees of freedom [DF] and P values) of temperature, grazing, fungus and their interactions on beech (*Fagus sylvatica*) wood decay rates (mg cm^−3^ d^−1^).

	F	DF	P
Temperature	19.7	1, 102	0.002
Grazing			
Fungus	6.7	2, 102	0.002
Temperature × Grazing			
Temperature × Fungus	16.6	2, 102	<0.001
Temperature × Grazing ×Fungus	2.4	6, 102	0.031

Non-significant (P ≥ 0.05) results are omitted for clarity.

## Discussion

A clear hierarchy of competitive dominance was evident at ambient temperature (16 °C) from three-species interactions between wood decomposer fungi (*P. velutina* > *R. bicolor* > *H. fasciculare*). Temperature elevated by 4 °C above ambient altered this hierarchy (supporting Hypothesis I) by stimulating the combative ability of *R. bicolor* such that it became dominant over *P. velutina*. The pattern of fungal dominance evident at elevated temperature supports previous work considering pair-wise interactions between the same three species in similar experimental systems [24]. This is in contrast to interactions in agar culture, where it is often not possible to predict multispecies interaction outcomes due to intransitive dominance hierarchies in pair-wise interactions (e.g. A > B, B > C, C > A; [13]). By promoting competitive dominance of the preferentially consumed species, warming also enabled grazing invertebrates to alter competitive interaction outcomes (supporting Hypothesis II). *Resinicium bicolor* is generally the most palatable to invertebrates, including those employed in the present study [21], [24]. Temperature elevation and grazing, therefore, exerted contrasting influences on fungal community development.

In contrast to Hypothesis (III) and previous work on two-species interactions [17], [24], the shift in competitive dominance at elevated temperature was not reflected in increased mycelial growth. Grazing by *O. asellus* affected mycelial extension; that by *R. bicolor* and *P. velutina* was reduced, enabling *H. fasciculare* to extend slightly (but not significantly) more rapidly. These results are analogous to research on plant communities, in which maximum community biomass was reached at relatively low species diversity levels [39] and grazing by vertebrate herbivores moderated responses to warming [4]. The marked effects of macrofauna on fungal growth and assemblage development supports the conclusion that they exert stronger grazing pressure on individual and interacting mycelia than smaller mesofauna [24], [25], even at low density [28]. The capacity for collembola to alter two-species interaction outcomes has been less consistent [17], [24]. In the present study, both collembola species influenced multispecies interaction outcomes, at elevated temperature. The stronger effect of woodlouse grazing on *P. velutina* (after removing the more palatable *R. bicolor*) enabled the weakest competitor, *H. fasciculare*, sometimes to gain territory.

Whilst top-down regulation of aboveground [40], [41] and bacterial-dominated belowground [42], [43] communities is well established, only recently has strong evidence emerged supporting the importance of this process in regulating the structure and function of fungal-dominated decomposer communities [44]. In the present study, increased capacity for top-down determination of the composition of a simple multispecies fungal community by grazing soil invertebrates is largely due to warming-induced promotion of the preferred fungal species. Elevated temperature may also have increased the size of collembola populations, as has previously been reported in microcosm studies with *R. bicolor* alone [16] and in two-species interactions [17]. The increased grazing pressure exerted by larger collembola populations [26]–[28] provides a greater potential to influence fungal species composition [17]. Macrofauna, including woodlouse and millipede, populations are also predicted to increase due to climate warming [45]. Mycophagous soil invertebrates have previously been hypothesised to drive, and respond to, changes in fungal community composition [14], [19]. The present and previous studies demonstrate that grazing by invertebrates is likely to be an important factor influencing the development, and response to climate change, of saprotrophic cord-forming fungal communities in woodland ecosystems.

Although fungal-mediated decomposition of colonised wood inocula was stimulated by elevated temperature, it was not competitively favoured mycelia that displayed the strongest responses (in contrast to Hypothesis III). Decomposition by *H. fasciculare* and *R. bicolor* was stimulated more by elevated temperature where their extra-resource mycelia were at a competitive disadvantage, due to either competitive weakness or selective grazing. The shift in fungal dominance hierarchy due to warming in ungrazed controls did, however, reduce resource decay by *P. velutina*. Species-specific decomposition rates changed, therefore, depending on mycelial competitive success. This suggests that changing species composition of saprotrophic macrofungi and mycophagous soil fauna could alter nutrient cycling and respiratory CO_2_ efflux [9]. For example, cellulolytic enzyme production and resource decay rates by *R. bicolor* are lower than *P. velutina* [46]. Grazers generally maintained multispecies assemblages at elevated temperature by preventing the competitive exclusion of a species from wood in the majority of cases. As a consequence, all fungal species continued to contribute to ‘community’ wide decomposition, regardless of competitive dominance on soil. The overall increase in fungal-mediated decomposition due to warming supports previous work in which grazers removed mycelial biomass, but did not prevent temperature-induced stimulation of resource decay [16], and has implications for the promotion of woodland soil nutrient turnover and CO_2_ efflux under climate change scenarios.

Although microcosm experiments provide mechanistic insights into otherwise complex and inaccessible ecological interactions, caution must be exercised in extrapolation to more natural conditions where biotic and abiotic variation are much greater [29], [30]. Mechanisms of interaction revealed by a microcosm approach will always demand further evaluation under natural conditions. The potential for grazing soil invertebrates to moderate climate-driven shifts in fungal species assemblages supports the argument that biotic and abiotic interactions are important buffers against community responses to climate change [47], [48]. Species losses could, therefore, have major implications for the insurance effect of biodiversity [49], [50], with functional group, rather than taxonomic, diversity being most important in soil decomposer systems [15], [51], [52].

## References

[1] Walther GR, Post E, Convey P, Menzel E, Parmesan C (2010) Ecological responses to recent climate change. Nature 416: 389–395. 10.1038/416389a 11919621

[2] Tylianakis JM, Didham RK, Bascompte J, Wardle DA (2008) Global change and species interactions in terrestrial ecosystems. Ecology Letters 11: 1351–1363. 19062363 10.1111/j.1461-0248.2008.01250.x

[3] Clark CM, Tilman D (2008) Loss of plant species after chronic low-level nitrogen deposition to prairie grasslands. Nature 451: 712–715. 18256670 10.1038/nature06503

[4] Post E, Pedersen C (2008) Opposing plant community responses to warming with and without herbivores. Proceedings of the National Academy of Sciences of the United States of America 105: 12353–12358. 18719116 10.1073/pnas.0802421105 PMC2527915

[5] Yang HJ, Wu MY, Liu WX, Zhang Z, Zhang N, Wan S (2011) Community structure and composition in response to climate change in a temperate steppe. Global Change Biology: 17: , 452–465.

[6] Bardgett RD, Freeman C, Ostle NJ (2008) Microbial contributions to climate change through carbon cycle feedbacks. The ISME Journal 2: 805–814. 18615117 10.1038/ismej.2008.58

[7] Singh BK, Bardgett RD, Smith P, Reay DS (2010) Microorganisms and climate change, terrestrial feedbacks and mitigation options. Nature Reviews Microbiology 8: 779–790. 20948551 10.1038/nrmicro2439

[8] Yuste JC, Penuelas J, Estiarte M, Garcia-Mas J, Mattana S, et al (2011) Drought-resistant fungi control soil organic matter decomposition and its response to temperature. Global Change Biology 17: 1475–1486.

[9] Hättenschwiler S, Tiunov AV, Scheu S (2005) Biodiversity and litter decomposition in terrestrial ecosystems. Annual Review of Ecology, Evolution and Systematics 36: 191–218.

[10] Baldrian P, Valaskova V (2008) Degradation of cellulose by basidiomycete fungi. FEMS Microbiology Reviews 32: 501–521. 18371173 10.1111/j.1574-6976.2008.00106.x

[11] Boddy L (1999) Saprotrophic cord-forming fungi: meeting the challenge of heterogeneous environments. Mycologia 91: 13–32.

[12] Fricker MD, Bebber D, Boddy L (2008) Mycelial networks: structure and dynamics. In: Boddy L, Frankland JC, van West P, editors. Ecology of saprotrophic basidiomycetes. London: Elsevier Ltd. pp. 3–18.

[13] Boddy L (2000) Interspecific combative interactions between wood decay basidiomycetes. FEMS Microbiology Ecology 31: 185–194. 10719199 10.1111/j.1574-6941.2000.tb00683.x

[14] A′Bear AD, Jones TH, Boddy L (2013) Potential impacts of climate change on interactions among saprotrophic cord-forming fungal mycelia and grazing soil invertebrates. Fungal Ecology doi: 10.1016/j.funeco.2013.01.009

[15] Gessner MO, Swan CM, Dang CK, McKie BG, Bardgett RD, et al (2010) Diversity meets decomposition. Trends in Ecology and Evolution 25: 372–380. 20189677 10.1016/j.tree.2010.01.010

[16] A′Bear AD, Boddy L, Jones TH (2012) Impacts of elevated temperature on the growth and functioning of decomposer fungi are influenced by grazing collembola. Global Change Biology 18: 1823–1832.

[17] Crowther TW, Littleboy A, Jones TH, Boddy L (2012) Interactive effects of warming and invertebrate grazing determine the outcomes of competitive fungal interactions. FEMS Microbiology Ecology 81: 419–426. 22432587 10.1111/j.1574-6941.2012.01364.x

[18] A′Bear AD, Crowther TW, Ashfield R, Chadwick DDA, Dempsey J, et al (2013) Localised invertebrate grazing moderates the effect of warming on competitive fungal interactions. Fungal Ecology 6: 137–140.

[19] Jones TH, Thompson LJ, Lawton JH, Bezemer TM, Bardgett RD, et al (1998) Impacts of rising atmospheric carbon dioxide on model terrestrial ecosystems. Science 280: 441–443. 9545223 10.1126/science.280.5362.441

[20] Pollierer MM, Langel R, Scheu S, Maraun M (2009) Compartmentalization of the soil animal food web as indicated by dual analysis of stable isotope ratios (^15^N/^14^N and ^13^C/^12^C). Soil Biology & Biochemistry 41: 1221–1226.

[21] Tordoff GM, Boddy L, Jones TH (2008) Species-specific impacts of collembola grazing on fungal foraging ecology. Soil Biology & Biochemistry 40: 434–442.

[22] Newell K (1984) Interaction between two decomposer basidiomycetes and a collembolan under Sitka spruce: distribution, abundance and selective grazing. Soil Biology & Biochemistry 16: 227–233.

[23] Newell K (1984) Interaction between two decomposer basidiomycetes and a collembolan under Sitka spruce: grazing and its potential effects on fungal distribution and litter decomposition. Soil Biology & Biochemistry 16: 235–239.

[24] Crowther TW, Boddy L, Jones TH (2011) Outcomes of fungal interactions are determined by soil invertebrate grazers. Ecology Letters 14: 1134–1142. 21929699 10.1111/j.1461-0248.2011.01682.x

[25] Crowther TW, Boddy L, Jones TH (2011) Species-specific effects of soil fauna on fungal foraging and decomposition. Oecologia 167: 535–545. 21562866 10.1007/s00442-011-2005-1

[26] Hanlon RDG, Anderson JM (1979) Effects of collembola grazing on microbial activity in decomposing leaf litter. Oecologia 38: 93–99. 28309073 10.1007/BF00347827

[27] Kaneko N, McLean MA, Parkinson D (1998) Do mites and Collembola affect pine litter fungal biomass and microbial respiration? Applied Soil Ecology 9: 209–213.

[28] Crowther TW, A′Bear AD (2012) Species-specific impacts of soil fauna on decomposer fungi are not masked by density-dependence. Fungal Ecology 5: 277–281.

[29] Lawton JH (1995) Ecological experiments with model systems. Science 269: 328–331. 17841248 10.1126/science.269.5222.328

[30] Lawton JH (1996) The Ecotron Facility at Silwood Park: the value of “big bottle” experiments. Ecology 77: 665–669.

[31] Dowson CG, Rayner ADM, Boddy L (1988) Inoculation of mycelial cord-forming basidiomycetes into woodland soil and litter. I. Initial establishment. New Phytologist 109: 335–341.

[32] Dowson CG, Rayner ADM, Boddy L (1988) Inoculation of mycelial cord-forming basidiomycetes into woodland soil and litter. II. Resource capture and persistence. New Phytologist 109: 343–349.

[33] IPCC (2007) Climate Change 2007: Synthesis Report. Contribution of Working Groups I, II and III to the Fourth Assessment Report of the Intergovernmental Panel on Climate Change [Core Writing Team, Pachauri RK, Reisinger A, editors]. Geneva: IPCC, 104 pp.

[34] Travleev AP (1960) The thermo-isolation effect of forest litter. Eurasian Soil Science 10: 1108–1110.

[35] Boddy L (1983) Microclimate and moisture dynamics of wood decomposing in terrestrial ecosystems. Soil Biology & Biochemistry 15: 149–157.

[36] Petersen H, Luxton M (1982) A comparative-analysis of soil fauna populations and their role in decomposition processes. Oikos 39: 287–388.

[37] Topp W, Kappes H, Kulfan J, Zach P (2006) Distribution pattern of woodlice (Isopoda) and millipedes (Diplopoda) in four primeval forests of the Western Carpathians (Central Slovakia). Soil Biology & Biochemistry 38: 43–50.

[38] R Development Core Team (2012) R: A language and environment for statistical computing. R Foundation for Statistical Computing, Vienna, Austria. URL:http://www.R-project.org .

[39] Tilman D, Knops J, Wedin D, Reich P, Ritchie M, Siemann E (1997) The influence of functional diversity and composition on ecosystem processes. Science 277: 1300–1302.

[40] Walker M, Jones TH (2001) Relative roles of top-down and bottom-up forces in terrestrial tritrophic plant-insect herbivore-natural enemy systems. Oikos 93: 177–187.

[41] Veen GF, Olff H, Duyts H, Van der Putten WH (2010) Vertebrate herbivores influence soil nematodes by modifying plant communities. Ecology 91: 828–835. 20426340 10.1890/09-0134.1

[42] Rønn R, McCaig AE, Griffiths BS, Prosser JI (2002) Impact of protozoan grazing on bacterial community structure in soil microcosms. Applied and Environmental Microbiology 68: 6094–6105. 12450833 10.1128/AEM.68.12.6094-6105.2002 PMC134433

[43] Moore JC, Mccann K, Setala H, De Ruiter PC (2003) Top-down is bottom up: does predation in the rhizisphere regulate aboveground dynamics? Ecology 84: 846–857.

[44] Crowther TW, Stanton D, Thomas S, A′Bear AD, Hiscox J, Jones TH, Voříšková J, Baldrian P, Boddy L (2013) Top-down control of soil fungal community composition by a globally distributed keystone consumer. *Ecology*, in press. 10.1890/13-0197.1 24400503

[45] David JF, Handa IT (2010) The ecology of saprophagous macroarthropods (millipedes, woodlice) in the context of global change. Biological Reviews 85: 881–895. 20412191 10.1111/j.1469-185X.2010.00138.x

[46] Crowther TW, Jones TH, Boddy L, Baldrian P (2011) Invertebrate grazing determines enzyme production by basidiomycete fungi. Soil Biology & Biochemistry 43: 2060–2068.

[47] Voigt W, Perner J, Jones TH (2007) Using functional groups to investigate community response to environmental changes: two grassland case studies. Global Change Biology 13: 1710–1721.

[48] Leutzinger S, Luo Y, Beier C, Dieleman W, Vicca S, Korner C (2011) Do global change experiments overestimate impacts on terrestrial ecosystems? Trends in Ecology and Evolution 26: 236–241. 21444122 10.1016/j.tree.2011.02.011

[49] Eklof JS, Alsterberg C, Havenhand JN, Sundback K, Wood HL, Gamfeldt L (2012) Experimental climate change weakens the insurance effect of biodiversity. Ecology Letters 15: 864–872. 22676312 10.1111/j.1461-0248.2012.01810.x

[50] Hooper DU, Adair EC, Cardinale BJ, Byrnes JEK, Hungate BA, et al (2012) A global synthesis reveals biodiversity loss as a major driver of ecosystem change. Nature 486: 105–108. 22678289 10.1038/nature11118

[51] Heemsbergen DA, Berg MP, Loreau M, van HalJR, Faber JH, Verhoef HA (2004) Biodiversity effects on soil processes explained by interspecific functional dissimilarity. Science 306: 1019–1020. 15528441 10.1126/science.1101865

[52] Crowther TW, Boddy L, Jones TH (2012) Functional and ecological consequences of saprotrophic fungus–grazer interactions. The ISME Journal 6: 1992–2001. 22717883 10.1038/ismej.2012.53 PMC3475375

